# Smoking and breast cancer

**DOI:** 10.1038/sj.bjc.6600933

**Published:** 2003-04-29

**Authors:** P Terry, K C Johnson

**Affiliations:** 1 Department of Epidemiology and Social Medicine, Albert Einstein College of Medicine, Bronx, NY 10461, USA; 2 Centre for Chronic Disease Prevention and Control, Population and Public Health Branch, Health Canada, Tunney's Pasture, P.L. # 0601C1, Ottawa, Ontario, Canada, K1A 0L2

**Sir**

The conclusion of the Collaborative Group on Hormonal Factors in Breast Cancer (Collaborative Group on Hormonal Factors and Breast Cancer, 2002) that cigarette smoking has little or no independent effect on the risk of developing breast cancer may not be warranted. Several recent studies of breast cancer risk suggest that a positive association with smoking may emerge 30–40 years after commencement (Terry and Rohan, 2002), particularly among chronic heavy smokers. There is also evidence to suggest that smoking prior to a first full-term pregnancy may be an important exposure (Russo *et al*, 1982; Band *et al*, 2002; Terry and Rohan, 2002). In addition, an increasing number of studies suggest that a possitive association between smoking and breast cancer risk may be stronger among (or limited to) women with certain genotypes (Terry and Rohan, 2002). To what extent the Collaborative Group's examination of smoking as ‘ever *vs* never’ or ‘current *vs* never’ could account for such possible time-dependent or genetic associations is unclear. The possibility of an association between cigarette smoking and breast cancer risk among certain groups of women should not be ruled out based solely on the Collaborative Group's analyses.
